# Detrimental pro-senescence effects of vitamin D on lung fibrosis

**DOI:** 10.1186/s10020-018-0064-z

**Published:** 2018-12-19

**Authors:** Trinidad Guijarro, Esmeralda Magro-Lopez, Joana Manso, Ricardo Garcia-Martinez, Maria Jesus Fernandez-Aceñero, Isabel Liste, Alberto Zambrano

**Affiliations:** 1 Functional Unit for Research into Chronic Diseases, Institute of Health Carlos III, Ctra. Majadahonda-Pozuelo Km 2, 28220 Madrid, Spain; 2 Hospital Clinico San Carlos, Madrid, Spain

## Abstract

**Background:**

The multiple biological effects of vitamin D and its novel activities on inflammation and redox homeostasis have raised high expectations on its use as a therapeutic agent for multiple fibrogenic conditions. We have assessed the therapeutic effects of 1α,25-Dihydroxyvitamin D_3_, the biologically active form of vitamin D, in the context of lung fibrosis.

**Methods:**

We have used representative cellular models for alveolar type II cells and human myofibroblasts. The extension of DNA damage and cellular senescence have been assessed by immunofluorescence, western-blot and senescence-associated β-galactosidase activity. We have also set up a murine model for lung fibrosis by intraperitoneal injections of bleomycin.

**Results:**

Vitamin D induces cellular senescence in bleomycin-treated alveolar epithelial type II cells and aggravates the lung pathology induced by bleomycin. These effects are probably due to an alteration of the cellular DNA double-strand breaks repair in bleomycin-treated cells.

**Conclusions:**

The detrimental effects of vitamin D in the presence of a DNA damaging agent might preclude its use as an antifibrogenic agent for pulmonary fibrosis characterized by DNA damage occurrence and cellular senescence.

## Introduction

Vitamin D is a hydrophobic secosteroid involved in the homeostasis of calcium, magnesium, iron, phosphate and zinc. The biological form of vitamin D (1α,25-Dihydroxyvitamin D_3_, from now on vitamin D) exerts its activity through the binding to the vitamin D receptor (VDR), a member of the nuclear hormone receptor superfamily (Pike and Christakos 2017). Besides its role on mineral metabolism, vitamin D regulates some chronic conditions including autoimmune, cardiovascular and respiratory diseases (Finklea et al. 2011). Vitamin D and its analogs are also active in the regulation of fibrosis that characterizes multiple chronic diseases such as renal, cardiac, liver or pulmonary fibrosis (Ding et al. 2013; Ito et al. 2013; Meredith et al. 2015; Zhang et al. 2015). Regarding the latter condition a preventive treatment with vitamin D supplementation ameliorated the severity of lung fibrosis probably due to its anti-inflammatory effects. However, the “therapeutic” role of vitamin D supplementation has not so far been assessed in the context of lung fibrosis. Idiopathic pulmonary fibrosis (IPF) is a form of progressive interstitial pneumonia of unknown etiology (King Jr. et al. 2011). IPF is an example of aging with median diagnosis at 66 years and estimated survival of 3–4 years. In essence, IPF pathogenesis is the consequence of an excessive matrix deposition leading to tissue scarring and irreversible organ injury probably due to a persistent input of damage and tissue repair response. A cellular state called senescence is implicated in the tissue repair program and in IPF physiopathology. Essentially, cellular senescence is an irreversible state of proliferation induced by multiple stressors such as oxidative stress, DNA damage, protein instability and telomere attrition. Its primary role is to make the cell damage inflicted visible for the immune system in order to draw the elimination of damaged cells. Multiple senescence biomarkers accumulate in both fibroblasts and epithelial cells in IPF lungs including the expression of CDKN1A (p21), CDKN2A (p16), senescence-associated β-galactosidase activity (SA-βgal) and DNA damage (Kuwano et al. 1996; Schafer et al. 2017). The occurrence of cellular senescence in IPF, in contrast to other fibrogenic conditions, has a detrimental role in the progression of the disease (Hecker et al. 2014; Lv et al. 2013; Shivshankar et al. 2012).

A number of evidences support the hypothesis that epithelial injury and impaired regeneration are sufficient to activate fibroblasts in the context of IPF. It has been proposed that cellular senescence induced by persistent epithelial damage may be the origin of such defective regeneration and the promotion of fibrosis. Indeed, accelerated epithelial senescence seems to play a role in the development of IPF (Aoshiba et al. 2013; Aoshiba et al. 2003; Chilosi et al. 2013; Kuwano et al. 2016). By other hand, vitamin D treatment or vitamin D receptor (VDR) have been implicated in the attenuation of fibrosis of various organs (Ding et al. 2013; Ito et al. 2013; Meredith et al. 2015; Zhang et al. 2015). Less is known, however, about its role in lung fibrosis. Some previous reports indicated that supplementation of vitamin D ameliorated the fibrotic effects induced by bleomycin (Zhang et al. 2015; Tan et al. 2016; Zhang et al. 2013). The common fact to these works is the preventive administration of vitamin D that, very probably, mediated these effects through its well-known anti-inflammatory properties.

We have evaluated the effects of the biological active form of vitamin D, 1α,25-Dihydroxyvitamin D_3_ or calcitriol, in two in vitro models for human alveolar type II (ATII) cells and myofibroblasts in terms of DNA damage, cellular senescence and fibrogenic activation. In addition, we have set up an IPF model consisting of the systemic exposure of mice to the antibiotic bleomycin in order to assess the potential “therapeutic” effect of vitamin D. The results indicate that only in the presence of the DNA damaging agent (bleomycin) vitamin D induces an alteration in the correct repair of DNA double-strand breaks (DSBs) and the induction of cellular senescence in epithelial cells. In our model of myofibroblasts, however, the response to bleomycin is the fibrogenic activation rather than their commitment to a senescence state. Moreover, our in vivo results indicate that rather than inhibiting lung fibrosis the treatment with vitamin D aggravates the disease probably due to the excessive presence of DNA damage in the form of DSBs especially in epithelial cells. Altogether our results suggest a detrimental role of vitamin D supplementation in lung fibrosis thus precluding its use as an antifibrogenic agent for IPF.

## Materials and methods

### Cell culture

Alveolar epithelial cells type II (A549, ATCC) and human myofibroblasts (Magro-Lopez et al. 2017) (Abyntek Biopharma) were maintained in DMEM medium supplemented with 10% FBS (Sigma), 2 mM glutamine and 100 U/mL of penicillin and streptomycin (Lonza). We used the active form of vitamin D (1α,25-Dihydroxyvitamin D_3_ or calcitriol; Sigma-Aldrich (cat.#D1530). Vitamin D stock was 10 μM in ethanol. Treatments were performed in cells maintained in DMEM supplemented with 10% hormone-depleted serum. This serum was prepared by using the anion exchange resin AG^R^1-X8 from BIO-RAD (cat.#1401441) as previously described (Zambrano et al. 2014). Bleomycin sulfate (cat.#CAYM13877–50) was purchased to VWR.

### Analysis of proteins by western-blot

Cell monolayers were washed with ice-cold PBS and lysed in triple-detergent lysis buffer [50 mM Tris-HCl pH 8.0, 150 mM NaCl, 0.02% sodium azide, 0.1% SDS, 1% NP-40, 0.5% sodium deoxycholate, 100 μg/ml PMSF, 2 μg/ml pepstatin, 2 μg/ml aprotinin, 2 μg/ml leupeptin, and phosphatase inhibitors cocktail 2 or 3 (cat.#P5726, P0044, Sigma-Aldrich)]. SDS-PAGE and immunoblotting were performed under standard conditions. Basically, samples in Laemmli buffer (30 μg/lane) were separated through 8% or 12% gels and transferred to nitrocellulose membranes for 90 min at RT in the presence of 20% methanol and 0,1% SDS. Membranes were blocked with 3% BSA in PBS-Tween 0,05% (PBST-BSA) and incubated O/N at 4 °C with specific antibodies diluted in PBST-BSA. Antibodies used were: γH2AFX (cat.#05–636, Millipore), CDKN1A (cat.#sc-6246, Santa Cruz Biotech), CDKN2A (cat.#04–239, Millipore), Cdkn2a (Millipore, cat.#PA5–20379), ACTA2 (cat.#MA5–11547, Thermoscientific), VIMENTIN (cat.#MA1–10459, Thermoscientific), Vitamin D receptor (VDR) (cat.#12550S, Cell Signaling), Tata box-binding protein (TBP) (cat.#818, abcam), tubulin (TUBA1A) (cat.#T9026, Sigma-Aldrich) and actin β (ACTB) (cat.#8224, abcam).

### Quantitative real-time RT-PCR (RT-qPCR)

RNA extraction and RT reactions were performed with Trizol reagent (cat.#15,596,026; Ambion) and the high-capacity cDNA kit (cat.#4,387,406; Applied Biosystems) following the manufacturer’s instructions. Real-time PCR was performed by using the powerUp SYBR Green mix (cat.#A25742) and the Quantstudio-3 system from Applied Biosystems. The relative amounts of the amplification products were calculated by the ΔΔCt method. The genes analyzed, and the sequences of the oligonucleotides employed in this study were the following:

**Table:** 

rRNA 18S	5’-GTAACCCGTTGAACCCCATT 5’-GCCTGCTTCAACCACCTTCTTG
*GAPDH*	5’-ACAGTCCATGCCATCACTGCC 5’-CCATCCAATCGGTAGTAGCC
*COLA1A* (Collagen type I)	5’-CAGCCGCTTCACCTACAGC 5’-TTTTGTATTCAATCACTGTCTTGCC
*COLA3A* (Collagen type III)	5′- ATGGTTGCACGAAACACACT 5′- CTTGATCAGGACCACCAATG
*ACTA2* (α-smooth muscle actin, α-SMA)	5′- CCGACCGAATGCAGAAGGA 5′- ACAGAGTATTTGCGCTCCGAA
*TGFB1*	5’-TGGCGATACCTCAGCAC 5’-CTCGTGGATCCACTTCCAG
*CYP24A1*	5’-GGTGACATCTACGGCGTAC 5’-CTTGAGACCCCCTTTCCAGAG
*Cyp24a1*	5’-CTGCCCCATTGACAAAAGGC 5’-CTAACCGTCGGGTCATCAGC

### ROS analysis

Reactive oxygen species were detected with the probes DA-DCFH (D6883, Sigma-Aldrich) and MitoSOX (M36008, Invitrogen) by flow-cytometry.

#### ROS detection by 2′-7′-dichlorofluorescein diacetate (DA-DCFH)

Cells growing in DMEM supplemented with 10% hormone-depleted serum were seeded at a density of 32,000 cells/cm^2^ in MW12 plates. At the following day the medium was replaced by fresh medium and the cells were pre-treated for 2 h with 5 nmol/L vitamin D (1α,25-Dihydroxyvitamin D_3_) and then treated for 24 h with bleomycin (12,5 μg/mL) and/or 5 nmol/L vitamin D. The cultures were then washed twice with Hank’s balanced salt solution (HBSS) and incubated for 1 h with HBSS. After that, the cells were incubated with DA-DCFH (20 μM) in HBSS for 1 h, washed and trypsinized. Trypsin was neutralized with HBSS-FBS 2% and the cells were collected and analyzed immediately by flow cytometry. The measurements were carried out using FACSCanto (BD Bioscience) cytometer. Cell debris as represented by distinct low forward and side scatter were gated out for analysis; 25,000 gated events for each condition were analyzed with the FACSCanto and Flow Jo software.

#### Detection of mitochondrial ROS by MitoSOX

The treatments were carried-out as for the DA-DCFH probe. Cells were incubated for 1 h with 5 μM MitoSOX in HBSS and then processed for flow cytometry.

The controls for ROS induction employed in these experiments consisted of treatments for 1 h with 50 μM terbutyl hydroperoxide (cat.#416,665, Sigma-aldrich).

### Senescence-associated β-galactosidase assay (SA-βgal)

SA-βgal assays were performed as previously described by (Dimri et al. 1995). Micrographs were taken in a microscope TS100F (Nikon) equipped with a digital camera DS-L1 (Nikon).

### Indirect immunofluorescence

Cells were seeded in 8-well chambers (cat.#154,534; Thermofisher Scientific) at a density of 20,000 cells/well. The following day the cells were treated as indicated in the corresponding experiments. Immunofluorescence was performed as previously described (Zambrano et al. 2014). Basically, cells were fixed in 2% PFA in PBS for 10 min at RT and permeabilised with 0.1% Triton X-100 and 0.1% sodium citrate (5 min/RT). Preparations were then washed with PBS and washing solution (PBS/0.25% BSA/0.1% Tween 20), blocked for 30 min with blocking solution (washing solution + 2.5% BSA), and incubated overnight with antibodies against TP53BP1 (NB-100-304, Novus Biologicals), PTK2 (focal adhesion kinase FAK; cat.#PA5–16676 Thermosicentific), ACTA2 (α-SMA; cat.#MA5–11547, Thermoscientific). Preparations were then washed with washing solution and incubated with secondary antibodies conjugated with alexa fluor dyes (488, 546) from Life Technologies (cat.#A-11029, cat.#A-11035) for 1 h at RT. Nuclei were counterstained with DAPI, and samples were mounted with ProLong Diamond (cat.#P36961; Life Technologies). Cell images were captured with a fluorescence microscopy (Zeiss Axio) equipped with a camera (Axiocam MRm) and AxioVision software. DNA damage foci were counted from > 150 cells for each experimental condition.

### Chromatin immunoprecipitation (ChIP) assays

Cells growing in DMEM supplemented with 10% hormone-depleted serum, were seeded in 150-mm dishes (6 × 10^6^ cells/plate) and the next day pretreated with 5 nmol/L of vitamin D (1α,25-Dihydroxyvitamin D_3_) for 2 h. Cells were then treated with bleomycin (12,5 μg/mL) for 6 h, washed and incubated in the presence or absence of 5 nmol/L vitamin D. After 48 h, cells were washed, and lysed following specifications of the magnetic-bead based ChIP Assay Kit (17–10,085, EMD Millipore), and sonicated in a Bioruptor (UCD-200TM; Diagenode). Immunoprecipitated DNA was purified with the QIAquick PCR purification kit (cat.#28,106, Qiagen). In each immunoprecipitation, 2–3× 10^6^ cells and 1 μg of the following antibodies: normal IgGs (cat.#sc-2027, sc-2025, Santa Cruz Biotech.), Histone H3 (cat.#ab1791; abcam), Acetylated Histone H3 (cat.#06–599; Upstate) and H3K27-3me (cat.#ab 6002; abcam) were used. DNA was amplified with primers P16(− 277): 5’-GTCCCTGCCCCTTTGCTATT and P16(+ 95): 5’-ACGGGTCGGGTGAGAGTC, that amplify a region encompassing the proximal P16 promoter (P16^INK4a^ promoter) at the human *CDKN2A* locus. Real-time PCR was performed by using the powerUp SYBR Green mix and the Quantstudio-3 system from Applied Biosystems. The analysis was performed by the percentage input method (Haring et al. 2007).

### Mice, bleomycin-based model of lung fibrosis and immunohistochemistry

Lung fibrosis was induced in pathogen-free 8- to 10-wk-old (weight 18–22 g) female C57BL/6 J mice by two intraperitoneal (i.p.) injections of bleomycin, diluted in 0,9% saline (0,1 mg/Kg b.w.), at days 0 and 2. At day 11, after the acute inflammatory response, the treatments with vitamin D (1α, 25-Dihydroxyvitamin D_3_) or vehicle were initiated (5 ng/ g b.w.). The doses employed and the protocol consisting of various i.p. injections have previously been described (Tan et al. 2016; Lee et al. 2015; Wang et al. 2016; Schapochnik et al. 2018). 1α,25-Dihydroxyvitamin D_3_ or the equivalent volume of vehicle was dissolved in saline immediately before the injections. Mice received an injection on days 11, 14, 17, 21 and 24 and were killed at day 28. The mice were divided randomly into the following experimental groups (five mice per group): a) saline only, b) bleomycin, c) bleomycin + vehicle and d) bleomycin + vitamin D (1α, 25-Dihydroxyvitamin D_3_). These experiments were repeated three times and were approved by the Committee of Bioethics and Animal Welfare of the Instituto de Salud Carlos III and Community of Madrid (file reference: PROEX-312). Protocols used followed the guidelines for animal protection reported by the Spanish national law RD 53/2013.

#### Histology

Lungs and liver were removed from each animal, dissected and fixed in 4% buffered formalin and embedded in paraffin wax or flash frozen with OCT compound. Four-micrometer sections from paraffin embedded tissue were stained with hematoxylin and eosin for assessment of tissue morphology or with trichrome to identify collagen.

#### Immunohistochemistry

Immunohistochemistry was performed on 4 μ-sections of paraffin-fixed embedded tissue. Antigen retrieval was performed with citrate buffer (pH 6) using a Microwave Tender Cooker (Nordic Ware) and a 700 W microwave (15 min 700 W/15 min 350 W). Endogenous peroxidase activity was inhibited with 0.3% H_2_O_2_ in methanol (25 min) and after that the sections were permeabilized and blocked simultaneously with PBS + 1%BSA + 0,1% TX-100, during 30 min. The slides were then washed once with PBS for 5 min. Endogenous biotin, biotin receptors and avidin binding sites were blocked by using the avidin/biotin blocking kit from Vector Laboratories (cat.#SP-2001). The slides were washed with PBS and incubated ON, in a humidity chamber, with the primary antibody diluted in blocking solution (PBS + 1%BSA). Incubations with the secondary biotinylated antibodies and development were performed with the kits MP-7402 (ImmPRESS) and SK-4605 (ImmPACT VIP HRP substrate) from Vector Laboratories, following their instructions. Methyl green (cat.#M8884-5G; Sigma-Aldrich) was used as counterstaining and xylene substitute mountant (cat.#1,900,231; Thermofisher) as mounting medium. Primary antibody used was γH2AFX (1:1000, cat.#05–636 Millipore). Damaged cells were counted from representative immunohistochemical fields for each experimental condition.

### Statistical analysis

Statistical significance of data was determined by applying a two-tailed Student’s t test or the analysis of variance followed by the Newman–Keuls or Bonferroni post-tests for the experiments with more than two experimental groups. *P* < 0.05 is considered significant. Significance of the analysis of variance post-test or the Student’s t test is indicated in the figures as *, *P* < 0.05; **, *P* < 0.01; and ***, *P* < 0.001. Statistics were calculated with the Prism 7 software (GraphPad Software). Data were subjected to the Shapiro-Wilk test and D’Agostino and Pearson omnibus test to verify their normality. The results presented in the figures are means ±SEM. Experiments were repeated at least two times.

## Results

### Effects of vitamin D and bleomycin in A549 cells and human lung fibroblasts

#### Effects of vitamin D in A549 cells [alveolar type II cells (ATII)]

**Fig. 1:**
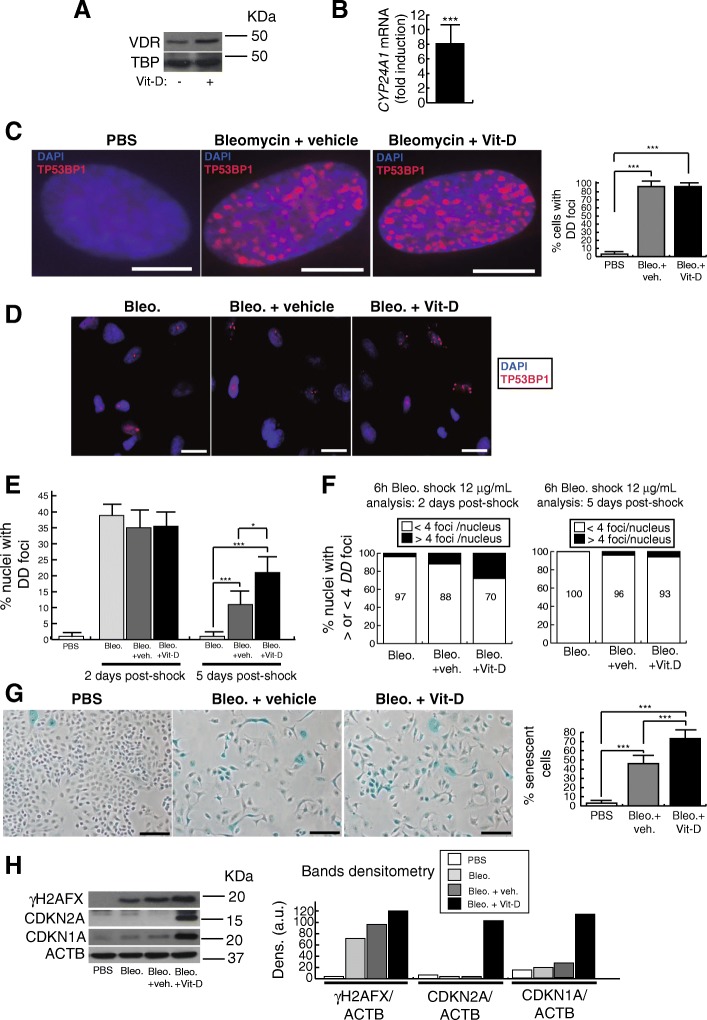
Effects of vitamin D and bleomycin in A549 cells. **a** Detection of vitamin D receptor (VDR) in A549 cell extracts. Cells were treated or not with 5 nmol/L vitamin D for 48 h. TBP (Tata box binding protein was used as loading control. KDa: kilodaltons. **b** RT-qPCR result: expression of *CYP24A1* in A549 cells treated for 48 h with 5 nmol/L vitamin D. Values are relative to A549 cells treated with vehicle. **c** Expression of DSBs induced by bleomycin. Representative micrographs of A549 nuclei harboring DNA damage foci containing TP53BP1, a marker of DSBs, are shown. Right panel: quantification of damaged cells. Scale bar: 5 μm. **d** Expression of TP53BP1 foci (red dots) in cultures of A549 cells pretreated with 5 nmol/L vitamin D for 2 h, subjected to a bleomycin shock (12 μg/mL for 6 h) and then treated with 5 nmol/L vitamin D or its vehicle. Representative micrographs taken at 48 h post-shock are shown. Scale bar: 20 μm. **e** Quantification of damaged cells (nuclei with TP53BP1 foci) at two and five days after bleomycin shock (12 μg/mL for 6 h). **f** Quantification of DD foci per nucleus at two and five days after bleomycin shock (12 μg/mL for 6 h). **g** Senescence-associated β-galactosidase assay (SA-βgal). Representative micrographs of A549 cultures at 48 h post-treatments are shown. Scale bar: 100 μm. Quantification of senescent cells is shown in the right panel. **h** Detection of γH2AFX, CDKN2A and CDKN1A in A549 cell extracts. Cells were pretreated with 5 nmol/L vitamin D for 2 h, subjected to a bleomycin shock (12 μg/mL for 6 h) and then treated with 5 nmol/L vitamin D or its vehicle. Cell extracts were obtained at 48 h post-shock. Control cultures were cells treated with bleomycin vehicle (PBS). ACTB was used as loading control. KDa: kilodaltons. Right panel: densitometry analysis of bands; a.u: arbitrary units

A549 cells express the VDR, which is overexpressed in the presence of 1α, 25-Dihydroxyvitamin D_3,_ vitamin D from now on (Fig. 1a). The treatment with 5 nmol/L vitamin D also increased the expression of vitamin D 24-hydroxylase (*CYP24A1*), a VDR target gene, indicating the VDR functionality in these cells (Fig. 1b). The treatment of A549 cells with 25 μg/mL of bleomycin for 48 h induced a drastic expression of DNA damage (DD) foci containing TP53BP1, a reliable marker of DNA double-strand breaks (DSBs) (Zambrano et al. 2014) (Fig. 1c). Under these conditions damaged cells (~ 100%) showed high densities of DD foci that precluded a reliable quantification of DD foci per nuclei however, the exposure of cells to a bleomycin shock (12 μg/mL for 6 h), allowed us to quantify the number of DD foci per nucleus after 2 and 5 days post-shock (Fig. 1d-f). At 2 days post-shock the percentage of damaged cells was similar in all three groups of bleomycin treated cells however, after 5 days post-shock, cells treated with vitamin D showed the highest levels of DNA damage (Fig. 1e). With regard to the amount of DD foci per nucleus, it was significantly higher in cells treated with both bleomycin and vitamin D compared to controls, at 2 days post-shock (Fig. 1f). At 5 days post-shock however, the majority of the cells exhibited < 4 foci per nucleus as a consequence of the cellular DNA repair activity (right panel, Fig. 1f).

**Fig. 2:**
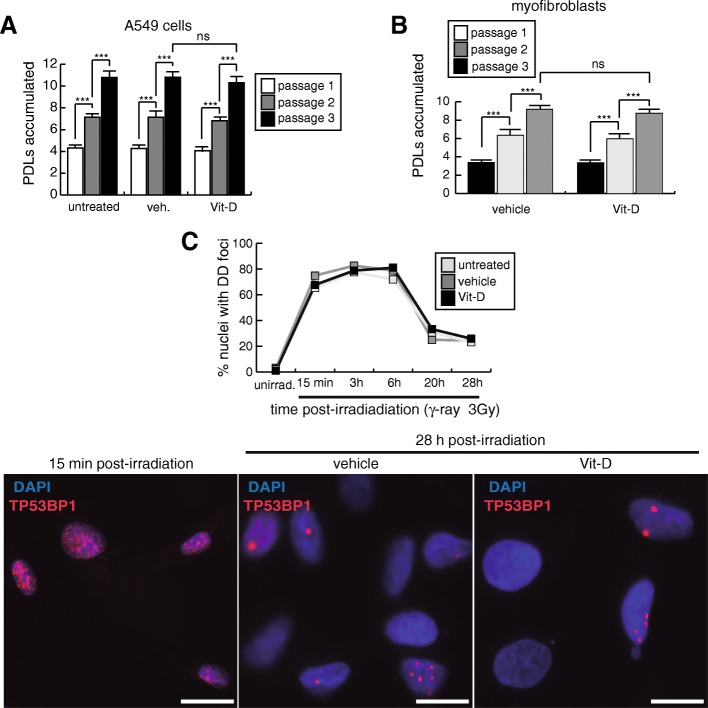
Results related to serial cell passages and γ-ray radiations. **a** Accumulation of population doubling levels during three passages of A549 cells treated with 5 nmol/L vitamin D or its vehicle. **b** Accumulation of population doubling levels during three passages of myofibroblasts treated with 5 nmol/L vitamin D or its vehicle. **c** Kinetics of DNA repair of DSBs induced by γ-radiation (3Gy). A549 cells were treated with 5 nmol/L vitamin D or its vehicle 2 h before irradiations and then subjected to 3Gy of γ-ray. At different times post-irradiation, cells were processed for indirect immunofluorescence of TP53BP1 to detect DSBs. DNA DD foci were quantified from representative micrographs (more than 150 cells examined). Bottom pictures show representative micrographs of the kinetics at 15 min and 28 h post-irradiation

As DNA damage is behind the onset of cellular senescence we evaluated the β-galactosidase activity (SA-βgal) in cultures treated for 48 h with 25 μg/mL of bleomycin. We consistently found significant higher levels of cellular senescence in cells treated with bleomycin and vitamin D compared to controls (Fig. 1g). The treatment of A549 with vitamin D in the absence of the damaging agent did not increase the low basal levels of DNA damage and senescence. Consistent with this, the continuous exposure of A549 cells to vitamin D during three consecutive passages did not alter their replication rates (Fig. 2a). The occurrence of cellular senescence was also evaluated by western-blot. Consistent with the increase in the levels of SA-βgal activity, we found higher expression levels of DNA damage and cellular senescence markers (γH2AFX, CDKN1A and CDKN2A) in cultures treated with bleomycin and vitamin D compared to controls (Fig. 1h).

#### Effects of vitamin D in human lung myofibroblasts

**Fig. 3:**
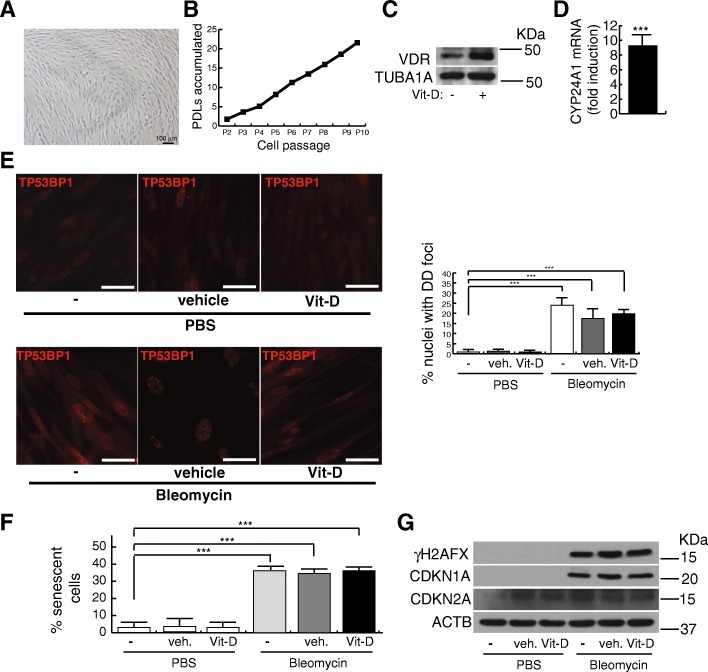
Effects of vitamin D and bleomycin in human myofibroblasts. **a** Representative micrographs of a typical myofibroblasts culture. Scale bar: 100 μm. **b** Accumulation of population doubling levels (PDLs) during ten consecutive passages. **c** Detection of vitamin D receptor (VDR) in myofibroblasts extracts. TUBA1A (tubulin α was used as loading control. KDa: kilodaltons. **d** RT-qPCR result: expression of *CYP24A1* in myofibroblasts cultures treated with 5 nmol/L vitamin D. Values are relative to cells treated with vehicle. **e** Expression of TP53BP1 foci (red dots) in cultures of myofibroblasts pretreated with 5 nmol/L vitamin D for 2 h, subjected to a bleomycin shock (12 μg/mL for 6 h) and then treated with 5 nmol/L vitamin D or its vehicle. Representative micrographs taken at 48 h post-shock are shown. Scale bar: 20 μm. Right panel: quantification of damaged cells (nuclei with TP53BP1 foci). **f** Quantification of the senescence levels (% of SA-βgal^+^ cells). Myofibroblasts were pretreated with 5 nmol/L vitamin D for 2 h, subjected to a bleomycin shock (12 μg/mL for 6 h) and analyzed at 48 h post-shock. PBS is the bleomycin vehicle; veh.: vitamin D vehicle. **g** Detection of γH2AFX, CDKN2A and CDKN1A in myofibroblasts cell extracts. Cells were pretreated with 5 nmol/L vitamin D for 2 h, subjected to a bleomycin shock (12 μg/mL for 6 h) and then treated with 5 nmol/L vitamin D or its vehicle. PBS is the bleomycin vehicle; veh.: vitamin D vehicle. ACTB was used as loading control. KDa: kilodaltons

**Fig. 4:**
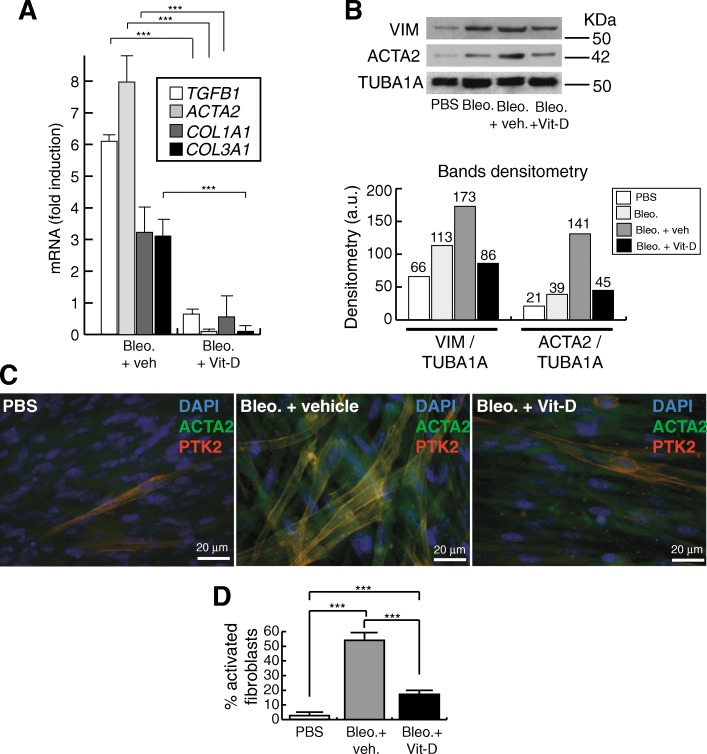
Effect of vitamin D on the fibrogenic activation of myofibroblasts. **a** RT-qPCR result: expression of a representative profibrogenic cluster of genes. Cells were pretreated with 5 nmol/L vitamin D for 2 h, subjected to a bleomycin shock (12 μg/mL for 6 h) and then treated with 5 nmol/L vitamin D or its vehicle. Analysis was performed 72 h post-shock. Values are folds of induction over control cultures (treated with bleomycin vehicle: PBS); veh.: vitamin D vehicle. The results presented in the figures are means ± SEM. Significance of the analysis is indicated as ***, *P* < 0.001. **b** Detection of VIM (vimentin) and ACTA2 (α-SMA) in myofibroblasts cell extracts. Cells were pretreated with 5 nmol/L vitamin D for 2 h, subjected to a bleomycin shock (12 μg/mL for 6 h) and then treated with 5 nmol/L vitamin D or its vehicle. Cell extracts were obtained at 72 h post-shock. PBS is the bleomycin vehicle; veh.: vitamin D vehicle. TUBA1A (tubulin α) was used as loading control. KDa: kilodaltons. Bottom panel: densitometry analysis of bands; a.u.: arbitrary units. **c** Representative micrographs of indirect immunofluorescence detection of ACTA2 (α-SMA fibers) and PTK2 (focal adhesion kinase), two markers of the profibrogenic activation. Cells were pretreated with 5 nmol/L vitamin D for 2 h, subjected to a bleomycin shock (12 μg/mL for 6 h) and then treated with 5 nmol/L vitamin D or its vehicle. Cells were processed for immunofluorescence 72 h post-shock. **d** Quantification of the immunofluorescence result (% of activated fibroblasts showing clear expression of α-SMA fibers and PTK2)

We also evaluated the effects of vitamin D in terms of DNA damage and fibrogenic activation in human lung myofibroblasts previously characterized (Magro-Lopez et al. 2017). These fibroblasts exhibit the typical spindle-shaped myofibroblasts morphology and can accumulate ~ 2–3 population-doubling levels (PDLs) per passage for at least 10 consecutive passages, consistent with the absence of replicative senescence (Fig. 3a, b). As for the case of A549 cells, these myofibroblasts express constitutively the VDR that together with the target gene *CYP24A1*, can be overexpressed in the presence of 5 nmol/L vitamin D (Fig. 3c, d). We performed this type of experiments in fibroblasts cultures with less than five passages where the levels of DNA damage and senescence in the population are consistently negligible. The treatment with bleomycin of fibroblasts cultures under the two conditions tested (either a constitutive exposure or in the form of a shock) induced the expression of TP53BP1-expressing DD foci that could not be further increased in the presence of vitamin D. In Fig. 3e the results corresponding to 6 h bleomycin shock (12 μg/mL) are shown. Contrary to the A549 cells, the treatment with vitamin D did not give rise to an increase in the levels of senescence and DNA damage reached by the sole exposure to bleomycin (Fig. 3f, g). The treatment with bleomycin for 3 days induced the expression of *TGFB1*, collagen I and III (*COL1A1, COL3A1*) and *ACTA2* [α-smooth muscle actin (α-SMA)], a representative cluster of genes whose expression is associated to the fibrogenic activation (Fig. 4a). As expected, the exposure to vitamin D inhibited significatively the expression of those profibrogenic genes and of VIMENTIN (VIM) (Fig. 4a, b). This inhibitory effect could also be observed by indirect immunofluorescence of α-smooth muscle actin fibers and the focal adhesion kinase (PTK2), reliable markers of the fibrogenic activation (Lagares et al. 2012) (Fig. 4c, d). In these cells, the exposure to bleomycin preferentially led to their fibrotic activation and, as previously described, vitamin D exhibited its antifibrogenic effects. In the absence of bleomycin, the vitamin D treatment did not alter either the expression of DNA damage or the levels of cellular senescence. Moreover, the continuous exposure of cells to vitamin D in the absence of bleomycin did not affect their replication rates (Fig. 2b).

### The increase in the levels of DNA damage induced by vitamin D in the presence of bleomycin is not mediated by ROS

**Fig. 5:**
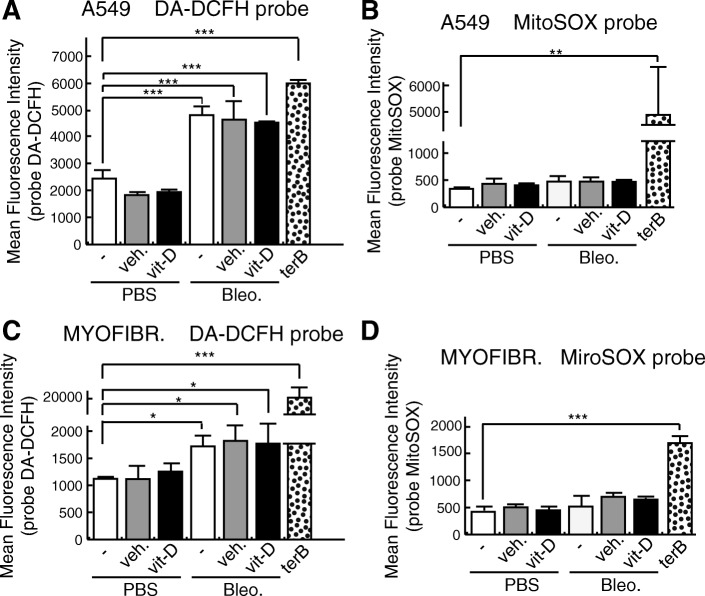
ROS analyses with DA-DCFH and MitoSOX probes. **a** Analysis of reactive oxygen species (ROS) by flow citometry of A549 cells (panels **a** and **b**) and myofibroblasts (panels **c** and **d**). Cells were pretreated for 2 h with 5 nmol/L vitamin D and then treated for 24 h with bleomycin (12,5 μg/mL) and/or 5 nmol/L vitamin D. The cultures were then washed twice with Hank’s balanced salt solution (HBSS) and incubated for 1 h with HBSS. After that, the cultures were incubated with DA-DCFH (20 μM) in HBSS for 1 h, washed and trypsinized. Trypsin was neutralized with HBSS-FBS 2% and the cells were collected and analyzed immediately by flow cytometry. The treatments with MitoSOX were carried-out as for the DA-DCFH probe. Cells were incubated for 1 h with 5 μM MitoSOX in HBSS and then processed for flow cytometry. Controls for ROS induction employed in these experiments consisted of treatment for 1 h with 50 μM terbutyl hydroperoxide (terB). The results presented in the graphs are means of the fluorescence intensity ± SEM. ANOVA result **a** (*P* < 0,001), ANOVA results **b**, **c** and **d** (*P* < 0,0001). Significance of the analysis post-test is indicated in the figure as *, *P* < 0.05; **, *P* < 0.01; and ***, *P* < 0.001

In the presence of Fe^2+^ and O_2_, bleomycin causes single-strand DNA breaks and DSBs. This occurs by chelation of the metal ion and the subsequent reaction with O_2_, that leads to the production of DNA-cleaving superoxide (O_2_^.-^) and hydroxide radicals (Bolzan and Bianchi 2018). We assessed whether vitamin D increase in DSBs in the presence of bleomycin could be the result of an increase in the levels of ROS. To do this, we evaluated both generic and mitochondrial ROS by flow cytometry and the probes DA-DCFH (2′,7′-dichlorodihydrofluorescein diacetate) and MitoSOX. DCFH-DA is a nonpolar dye, converted into the polar derivative DCFH by cellular esterases that are nonfluorescent but switched to highly fluorescent DCF when oxidized by intracellular ROS and other peroxides. MitoSOX is a fluorogenic dye that targets the mitochondria and is rapidly oxidized by superoxide but not by other ROS or reactive nitrogen species generating systems. In both type of cells, the treatment with bleomycin led to an elevation in ROS levels when the probe DCFH-DA was employed although fibroblasts seemed to be more resistant to bleomycin-induced ROS (Fig. 5a, c). However, this bleomycin-mediated induction of ROS did not modify the redox status of MitoSOX (Fig. 5b, d). In any case, vitamin D treatment increased the levels of ROS compared to its controls (Fig. 5a-d) according to previous reports supporting the notion that vitamin D has roles as antioxidant and antiaging (Berridge 2016; Datta Mitra et al. 2013; Jain and Micinski 2013; Valcheva et al. 2014). Next, we evaluated the cellular repair potential by irradiating A549 cells with γ-ray in the absence of bleomycin. Cells treated with vitamin D and subjected to 3Gy of γ radiation showed no alterations in the cellular DSBs repair capabilities (Fig. 2c).

### The increase in the levels of CDKN2A induced by vitamin D in the presence of bleomycin is due to a posttranscriptional mechanism

**Fig. 6:**
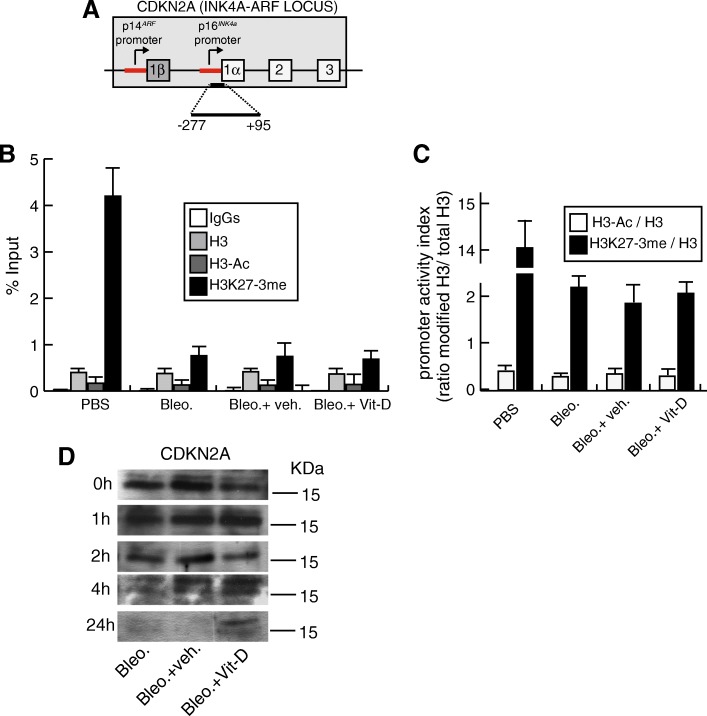
Chromatin immunoprecipitation (ChIP) analysis of the *CDKN2A* (p16^*INK4a*^) proximal promoter. **a** Diagram (not to scale) that illustrates the *CDKN2A* (*INK4A-ARF*) locus showing its exon/intron structure and the region analyzed corresponding to the proximal p16^*INK4a*^ promoter (region − 277 to + 95). **b** ChIP result (% of input) obtained with normal IgGs, and the antibodies directed against histone H3 (H3), acetylated histone H3 (H3-Ac) and Histone H3 trimethylated in Lys27 (H3K27-3me). Cells were pretreated with vitamin D or its vehicle, subjected to a bleomycin shock (12 μg/mL for 6 h) and then treated with 5 nmol/L vitamin D or its vehicle. Control cultures correspond to cells treated with bleomycin vehicle (PBS). **c** Promoter activity index as determined by the ratio of the % input of modified histone H3/ % input of total histone H3. Transcriptional activation is determined by the ratio H3Ac/H3 and repression by the corresponding ratio H3K27-3me/H3. The results presented in the graphs are means ±SEM. ANOVA *P* < 0,0001. **d** Determination of CDKN2A protein half-life. Cells were pretreated with 5 nmol/L vitamin D or its vehicle, subjected to a bleomycin shock (12 μg/mL for 6 h) and then treated with 5 nmol/L of vitamin D or vehicle for 24 h. After that the cells were incubated with 100 μM of cycloheximide for 24 h. Detection of CDKN2A protein was performed in cell extracts of cultures released from cycloheximide inhibition at different times (from 0 to 24 h). KDa: kilodaltons

The onset of cellular senescence observed in A549 treated with bleomycin and vitamin D cells was consistently associated with DNA damage and an increase in the expression levels of CDKN1A and CDKN2A. The augmented expression of these two senescence markers was not associated with an increase in the levels of mRNA (data not shown) suggesting a posttranscriptional regulatory mechanism established by vitamin D. To confirm this hypothesis, we first confirmed the absence of transcriptional activity of the *CDKN2A* promoter upon the treatment with bleomycin and vitamin D. In proliferating cells *CDKN2A* locus is maintained inactive by the continued presence of the histone methyltransferase EZH2-containing polycomb protein complex and it has been proposed that this is achieved by an enrichment of the repressive mark H3K27-3me (histone H3 trimethylated on Lys27) (Ito et al. 2018; Jacobs et al. 1999). In response to multiple stress and senescing signals the activity of EZH2 and the levels of H3K27-3me decreases giving rise to transcriptional activation. We therefore analyzed the presence of the repressive H3K27-3me mark and of chromatin activation (acetylated histone H3; H3-Ac) at the proximal *CDKN2A* promoter (− 277 to + 95) by chromatin immunoprecipitation (Fig. 6a). *CDKN2A* promoter from cultured cells in the absence of bleomycin is enriched in H3K27-3me consistent with the absence of *CDKN2A* expression. As expected, the treatment with bleomycin led to a drastic decrease in the repressive mark concomitant with the induction of senescence. However, in the presence of the damaging agent, neither the repressive mark nor the activation mark (H3-Ac) was increased with vitamin D (Fig. 6b). As a consequence, the promoter activity index determined as the ratios of H3-Ac/total H3 and H3K27-3me/total H3 was maintained unaltered under the presence of bleomycin (Fig. 6c). Thus, the increase in the CDKN2A expression observed upon vitamin D treatment was not associated to transcriptional activation. We next evaluated the possibility that vitamin D treatment in the presence of bleomycin could increase the CDKN2A protein half-life. To do this, we analyzed CDKN2A expression at different times post-treatment with the inhibitor of protein synthesis cycloheximide. As shown in Fig. 6d, only in cells treated with vitamin D and bleomycin significant levels of CDKN2A protein could be detected after 24 h of the cycloheximide release.

### Vitamin D treatment aggravates bleomycin-induced lung fibrosis by increasing DNA damage

**Fig. 7:**
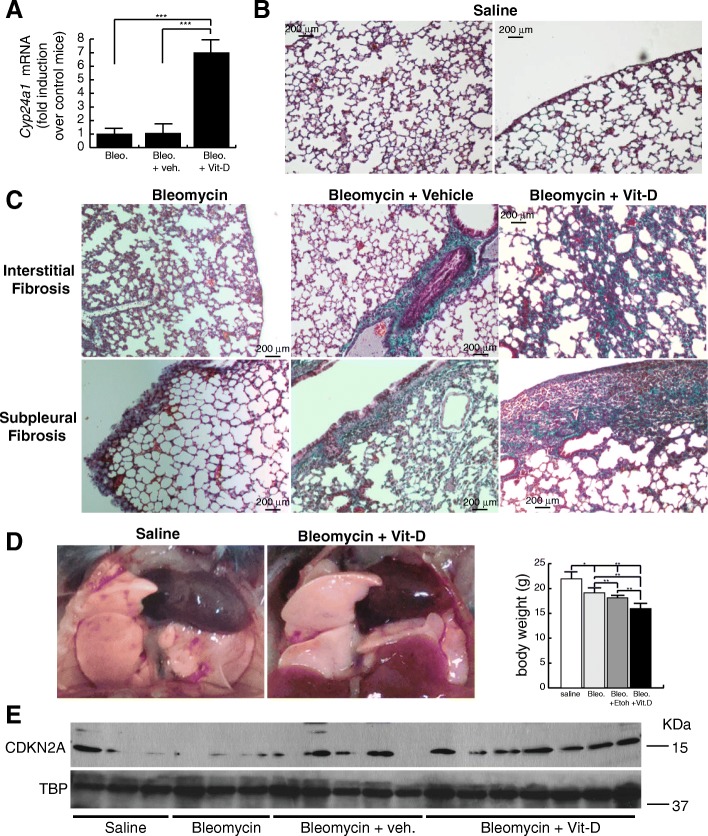
Vitamin D exacerbates lung fibrosis in mice. **a** Expression of liver *Cyp24a1*. Values are folds of induction over control mice (treated with bleomycin vehicle: saline); veh.: vitamin D vehicle. The results presented in the figures are means ± SEM. Significance of the analysis is indicated as ***, *P* < 0.001. **b** Representative micrographs of Masson’s stain of lung sections from control mice (treated with bleomycin vehicle: saline). Scale bar: 200 μm. **c** Representative micrographs of Masson’s stains of lung sections of mice treated with bleomycin and vitamin D or its vehicle. Scale bar: 200 μm. **d** Representative macrographs of lungs from control mice and treated with bleomycin and vitamin D. Right panel: body weights of a representative experimental group of mice (*n* = 20). The results presented in the graphs are means ± SEM. ANOVA result (*P* < 0,01). Significance of the analysis post-test is indicated in the figure as *, *P* < 0.05 and **, *P* < 0.01. **e** Expression levels of CDKN2A from lung tissue homogenates of representing the four experimental groups. TBP (tata binding protein) was used as loading control

**Fig. 8:**
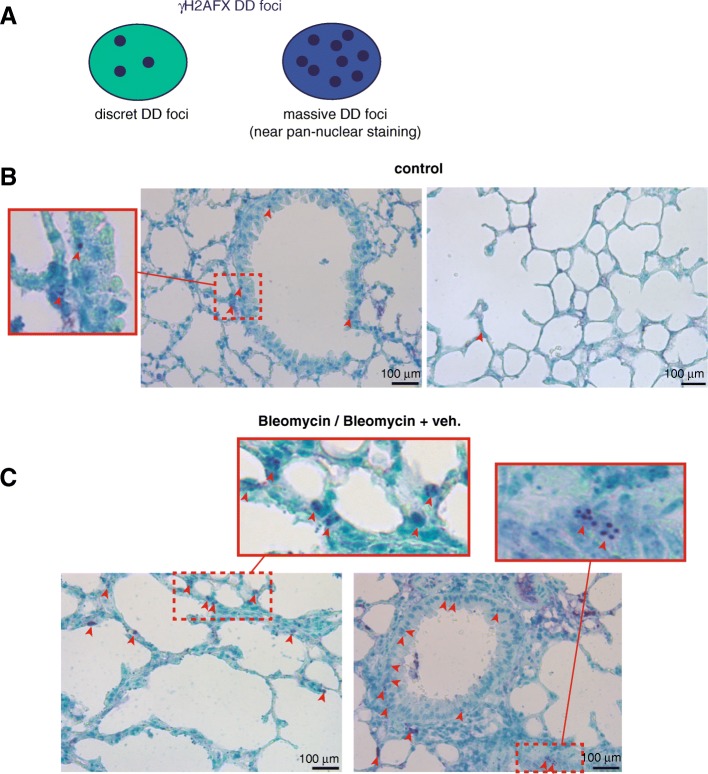
DNA damage expression in lung tissue: expression of γH2AFX foci. **a** Illustration showing the staining pattern of γH2AFX. γH2AFX is typically expressed as discrete foci or as a near pan-nuclear staining when the occurrence of DD foci is massive. **b** Expression of γH2AFX foci in representative sections of lung tissue from control mice (treated with saline). A magnification of an image of a respiratory bronchiole is depicted in the square on the left. Arrowheads signal some of the nuclei harboring DD foci containing γH2AFX distributed throughout the respiratory bronchiole or in alveolar fields (photograph on the right). **c** Expression of γH2AFX foci in representative sections of lung tissue from mice treated with bleomycin or bleomycin + vehicle. Two magnifications of the images are depicted in the rectangles on the top. Arrowheads signal some of the nuclei harboring some DD foci containing γH2AFX indicative of severe DNA damage. The magnitude and distribution of the damage observed was very similar in both groups. Scale bars: 100 μm

**Fig. 9:**
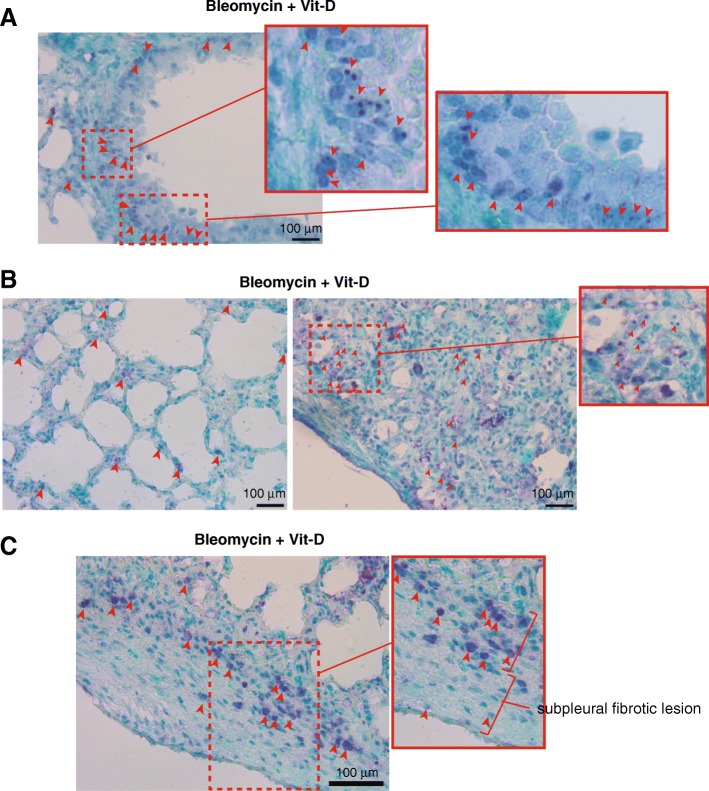
Exacerbation of the DNA damage expression in lung tissue from mice treated with bleomycin and vitamin D. **a** Representative micrograph of a bronchiole surrounded by a fibrotic focus. Two magnifications of the image are depicted in the rectangles on the right. Arrowheads signal some of the multiple nuclei harboring DD foci containing γH2AFX indicative of severe DNA damage. **b** Representative micrographs of interstitial fibrosis (left) and of an area abnormally re-epithelialized (right) showing the expression of massive DNA damage specially in the epithelial component. **c** Representative image of a subpleural fibrotic lesion lined by an abnormally re-epithelialized area showing the presence of severe DNA damage especially in epithelial cells. Scale bars: 100 μm

In order to build an adequate model for IPF in mice we treated C57BL/6 mice with two intraperitoneal injections of bleomycin (100 mg/Kg b.d.) and after 11 days from the second injection we started the “therapeutic” treatment with vitamin D (5 ng/ g b.w.), or its vehicle, for 2 weeks. After that, at day 28, animals were sacrificed and examined. Mice were randomly divided into four groups: control, bleomycin, bleomycin+vehicle and bleomycin+vitamin D (3 experiments; *n* = 60). We first validated vitamin D functionality by assessing the expression of liver *Cyp24a1* by RT-qPCR. As shown in Fig. 7a, mice treated with bleomycin and vitamin D showed, as expected, the highest expression levels of this VDR target gene. We next analyzed the extension of fibrosis by the Masson’s trichrome stain. We scored the levels of fibrosis as 0 (no fibrosis), 1 (low fibrosis), 2 (mild fibrosis) and 3 (severe fibrosis). We found that mice treated with bleomycin alone or with vitamin D vehicle consistently showed fibrosis levels 1–2, characterized by interstitial fibrosis and fibrotic foci or patches along the pleura (subpleural fibrosis). However, mice treated with bleomycin and vitamin D showed the highest levels of fibrosis characterized by a severe architectural distortion and a striking subpleural fibrosis (Fig. 7b, c). Compared to controls mice, lungs from animals treated with bleomycin and vitamin D exhibited a characteristic whitish color probably due to the extension of fibrotic scarring (Fig. 7d). An additional striking feature of these mice was the weight loss during the progression of the disease. Mice treated with bleomycin and vitamin D exhibited the highest loses of weight concomitant with the exacerbation of lung pathology (right panel Fig. 7d). We also evaluated the expression of *Cdkn2a* by western-blot of tissue homogenates from representative experimental samples. *Cdkn2a* expression was significatively elevated in mice treated with bleomycin and vitamin D (Fig. 7e) compared to the rest of the groups. We next evaluated the expression of DNA damage by immunohistochemical stain of a marker or DNA damage and cellular senescence (Zambrano et al. 2014; Sedelnikova et al. 2004). The staining pattern of γH2AFX antibody consisted of either discrete DD foci corresponding to DSBs or massive nuclear accumulation of these DD foci showing a near pan-nuclear staining (Fig. 8a). Lung tissue from control mice showed low levels of DNA damage (< 5% of the cells) consisted primarily in cells harboring a few DD foci. We found a homogeneous distribution of DNA damage at bronchiolar epithelia and alveolar cells throughout the lung (Fig. 8b). Bleomycin or bleomycin + vehicle mice showed higher levels of DNA damage (10–20% of the cells) compared to control mice. This damage consisted of cells, mainly epithelial cells throughout the parenchyma, harboring several DD foci (Fig. 8c). The fibrosis extension in bleomycin + vitamin D treated animals was strikingly exacerbated exhibiting architectural distortion and subpleural fibrosis. This exacerbation correlated well with the expression of DNA damage. Damaged cells were more abundant in this group (> 20% of the cells) than in the others, exhibiting nuclei with a large amount of DD in bronchiolar epithelium or at the alveolar fields (Fig. 9a, b). We also observed the typical abnormal re-epithelization of lesions found in IPF lungs with the presence of damaged cells (right picture, Fig. 9b). In addition, we found a differential distribution of damaged cells specifically at subpleural lesions. Epithelial cells in and around central and subpleural fibrotic foci harbored significant higher levels of DD foci compared with fibroblasts from the fibrotic lesions (Fig. 9c) in concordance with the in vitro fibroblasts resistance to a damage insult.

## Discussion

We have analyzed the effects of the biological active form of vitamin D (calcitriol or 1α, 25-Dihydroxyvitamin D_3_) on representative cellular models for ATII cells and myofibroblasts, in terms of DNA damage, cellular senescence and fibrogenic activation. We have also assessed its potential antifibrotic therapeutic role in a mouse model of IPF based on intraperitoneal injections of bleomycin.

Our results indicate that low concentrations of bleomycin induce DSBs and cellular senescence more efficiently in A549 cells. In a previous report, Aoshiba et al. also described that immortalized A549 cells rapidly become senescent upon bleomycin treatment (Aoshiba et al. 2003). In the presence of a DNA damaging agent, the decrease in DNA damage expression as a consequence of DNA repair was lower in vitamin D treated cells than in the control ones, suggesting an alteration in the cellular DSBs repair capabilities of A549 cells. This alteration would only take place in cells treated with bleomycin as cells treated with vitamin D in the absence of bleomycin do not exhibit more DNA damage than the controls and cells treated with vitamin D and subjected to 3Gy of γ radiation showed no alterations in the cellular repair capacities of DSBs, at least, at the times assessed.

Vitamin D receptor is expressed in both cell types and fully functional as determined by the ligand induction of *CYP24A1* expression. Neither the replication rates nor the ROS levels were increased in cells exposed to 5 nmol/L vitamin D. However, under the presence of a DNA damaging agent, vitamin D induced a posttranscriptional induction of *CDKN2A* in A549 cells that together with the expression of DNA damage and the SA-βgal activity contributed to the establishment of a canonical DNA damage-induced senescence program or premature senescence.

Bleomycin causes fibrotic lesions in C57BL/6 mice within a short period of time. It has been reported that the main histological hallmarks of IPF are present in bleomycin treated animals but it largely depends on the route of bleomycin administration (Chua et al. 2005). For instance, intratracheal instillation, the standard route of administration, produces a bronchiocentric fibrosis, whereas intravenous or intraperitoneal administration induces subpleural scarring similar to IPF (Chua et al. 2005). Bleomycin rapidly induces an acute inflammatory response lasting up to 8 days followed by the pure fibrotic response consisting of matrix deposition and lung architecture distortion out to 28 or 35 days (Moeller et al. 2008). Assuming this sequence of events, treatments during the first 7 days can be considered as preventive while treatments during the later stages after day 7–10 can be considered as therapeutic (Moeller et al. 2008). Some reports have indicated that supplementation of vitamin D ameliorated the fibrotic effects induced by bleomycin (Zhang et al. 2015; Tan et al. 2016; Zhang et al. 2013) but these effects were probably due to a preventive, anti-inflammatory treatment of vitamin D.

Bleomycin exposure of myofibroblasts however resulted in a fibrogenic activation that could be inhibited by the treatment with vitamin D as previously described. This differential response to DNA damage might be due to an inherent resistance of myofibroblasts to bleomycin ROS-induced DNA damage. A growing body of evidence indicates that epithelial fibroblast interactions play an essential role in the development of IPF (Noble 2008; Selman and Pardo 2006; Yanagi et al. 2015). Two types of AECs line up the alveolar wall, ATI and ATII cells. As result of the persistent tissue damage there is a net loss of alveolar epithelium with the subsequent integrity loss of the basement membrane. ATII cells are more resistant than ATI cells to damage and become activated to stimulate fibroblasts to produce extra cellular matrix components. Our results support this notion. Severely damaged cells with a phenotype compatible with ATII cells are lining the alveoli and in areas of abnormal re-epithelization. In addition, epithelial cells from bronchioles show clear signs of DNA damage in contrast to myofibroblasts from fibrotic foci such as those embedded at subpleural foci. These observations suggest that epithelial cells assimilate the bulk of the damage inflicted and trigger the activation and proliferation of fibroblasts.

These findings indicate vitamin D exacerbates the occurrence of fibrosis probably by increasing DNA damage primarily in epithelial cells of lungs subjected to a bleomycin insult. The presence of DNA damage and probably the onset of cellular senescence in ATII cells would compromise its function in the alveolar homeostasis maintenance. This abnormal alveolar regeneration has been observed in lungs from patients with IPF (Noble 2008; Selman and Pardo 2006; Kawanami et al. 1982).

Although some epidemiology studies suggest a potential relationship between vitamin D deficiency and advanced lung disease (Forli et al. 2004; Mascitelli et al. 2010), a systematic review of studies related to health outcome and vitamin D intakes shows no relationship between vitamin D supplement and the reduction in overall mortality of any cause (Chung et al. 2009; Manson et al. 2018; Newberry et al. 2014). Our study suggests that vitamin D supplementation could have an adverse effect in patients with signs of lung fibrosis.

## Conclusions

Vitamin D exhibited an unexpected detrimental activity in the presence of a DNA damaging agent. This activity is probably due to a suboptimal capability repair of DSBs associated to the establishment of a senescence phenotype of ATII cells and to the exacerbation of the lung pathology induced by bleomycin. The detrimental effects of vitamin D in the presence of a DNA damaging agent might preclude its use as an antifibrogenic agent in pulmonary fibrosis characterized by DNA damage occurrence and cellular senescence such as the idiopathic pulmonary fibrosis.

## Abbreviations

ACTA2: Actin, alpha 2, smooth muscle, aorta (α-smooth muscle actin, α-SMA)
ACTB: Actin beta
AEC: Alveolar epithelial cell
ANOVA: Analysis of variance
ATI: Alveolar type I cell
ATII: Alveolar type II cell
b.w.: Body weight
BSA: Bovine serum albumin
CDKN1A: Cyclin dependent kinase inhibitor 1A (p21)
CDKN2A: Cyclin dependent kinase inhibitor 2A (p16)
COL1A1: Collagen type I alpha 1 chain
COL3A1: Collagen type III alpha 1 chain
CYP24A1: Cytochrome P450 family 24 subfamily A member 1
DD: DNA damage
DSBs: DNA double-strand breaks
FBS: Fetal bovine serum
H2AFX: H2A histone family member X
IgGs: Immunoglobulin G
MW6: 6-well plates
O/N: Over night
PBS: Phosphate buffered saline
PTK2: Focal adhesion kinase (FAK)
ROS: Reactive oxygen species
SA-βgal: Senescence associated β-galactosidase activity
SDS: Sodium dodecyl sulfate
SEM: Standard error of the mean
TGFB1: Transforming growth factor beta 1
TP53BP1: Tumor protein p53 binding protein 1
TUBA1A: Tubulin alpha 1a
VDR: Vitamin D receptor
VIM: Vimentin
μm: Micrometer
